# Development and External Evaluation of Hypoxia-Related Prognostic Signatures in Head and Neck Squamous Cell Carcinoma

**DOI:** 10.3390/genes17101256

**Published:** 2026-10-11

**Authors:** Jimmy Carter Osei, Abdullah Aziz, Tim Andrew Davies Smith

**Affiliations:** 1 School of Computer Sciences and Engineering, Bangor University, Bangor LL57 1UT, UK; jms24xfh@bangor.ac.uk (J.C.O.); bdz25ynl@bangor.ac.uk (A.A.); 2 Division of Cancer Sciences, University of Manchester, Manchester M20 4GJ, UK

**Keywords:** HNSCC, hypoxia, radiotherapy, gene expression signature, prognosis

## Abstract

Background: We evaluated breast cancer-derived hypoxia and radiosensitivity signatures in head and neck squamous cell carcinoma (HNSCC), then developed and externally evaluated HNSCC prognostic signatures from hypoxia-related genes. Methods: This retrospective study used TCGA HNSC for development (514 patients; 217 deaths) and GSE65858 for external evaluation (270 patients; 94 deaths). LASSO Cox regression selected models from 414 hypoxia-related genes measurable in both cohorts. The 32-gene primary model and 13-gene sensitivity model were locked before external survival testing. External analyses assessed continuous scores, with additional adjustment for age, stage, tumour site, HPV DNA status and smoking. Cross validation with standardisation within training folds and bootstrap gene selection stability were examined in subsequent checks. Results: The main analyses did not establish prognostic associations for the breast-derived signatures in HNSCC. The primary HNSCC model had a mean development cross-validation C index of 0.628. In GSE65858, univariable hazard ratios per score standard deviation were 1.29 (95% confidence interval 1.05 to 1.59; *p* = 0.016) for the primary model and 1.28 (1.03 to 1.59; *p* = 0.024) for the sensitivity model. Their C indices were 0.566 and 0.581. After clinical adjustment in 265 patients with 92 deaths, the corresponding hazard ratios were 1.24 (0.99 to 1.55; *p* = 0.065) and 1.20 (0.95 to 1.51; *p* = 0.117). Neither signature reached statistical significance in the analyses using detailed HPV information. Five-year median-split log-rank *p* values were 0.095 and 0.204. Conclusions: The signatures showed unadjusted associations with overall survival in an external cohort, but discrimination was modest and independent prognostic value was not established after clinical adjustment. These exploratory prognostic signatures require further evaluation alongside clinical factors. The findings do not establish a quantitative measure of tumour hypoxia or utility for treatment selection.

## 1. Introduction

Hypoxia-related gene expression has been investigated as a biomarker of prognosis and treatment response in head and neck squamous cell carcinoma (HNSCC). Winter and colleagues developed a hypoxia metagene and examined its association with outcome in head and neck cancer [1]. Toustrup and colleagues developed a 15-gene classifier to identify tumours for which hypoxic modification of radiotherapy could be beneficial [2]. These approaches address related but distinct questions: whether a tumour expression profile is associated with subsequent outcome, and whether it identifies differences in benefit from a particular treatment.

The transfer of a signature between cancer types requires separate evaluation. Eustace and colleagues reported that a 26-gene hypoxia signature predicted benefit from hypoxia-modifying treatment in laryngeal cancer, but did not establish the same predictive value in bladder cancer [3]. This finding motivates testing a signature in the cancer type in which it was derived rather than assuming that its performance is the same across cancer types. For a prognostic model, a corresponding question is whether the association with survival persists when its fixed gene weights are applied to a separate patient cohort.

Existing tumour gene expression datasets provide a route to signature development without new laboratory experiments. In bladder cancer, Yang and colleagues derived a 24-gene hypoxia signature from publicly available transcriptomic data and subsequently evaluated it in the BCON trial [4]. Smith and colleagues later examined this signature in BC2001 and BCON, reporting exploratory evidence that higher hypoxia scores were associated with poorer outcomes following hypofractionated radiotherapy. Fractionation schedules were not randomised, and prospective evaluation was recommended before using the score to guide treatment [5]. These studies motivate the development and independent evaluation of tumour-specific signatures using existing data, while emphasising that prognostic associations and prediction of treatment benefit require separate assessment.

Our previous breast cancer study developed a three-gene hypoxia score and a four-gene DNA repair radiosensitivity score using TCGA BRCA, with further prognostic evaluation of the hypoxia score in METABRIC [6].

This study first evaluated the breast-derived signatures in TCGA HNSC and then developed an HNSCC prognostic model using a candidate pool assembled from published hypoxia gene sets. TCGA HNSC was used for model development, and GSE65858 for external evaluation [7,8]. The primary objective of the model development component was to assess the association between the fixed signature and overall survival in the external cohort. A smaller model was evaluated as a sensitivity analysis, and adapted published hypoxia scores provided additional comparisons. The external evaluation addressed prognosis; it did not test whether the new signatures predicted benefit from radiotherapy or hypoxia-modifying treatment.

## 2. Materials and Methods

### 2.1. Study Design and Cohorts

This retrospective study used publicly available tumour gene expression and clinical data from two cohorts of patients with head and neck squamous cell carcinoma (HNSCC). RNA sequencing data from The Cancer Genome Atlas HNSC project (TCGA HNSC) were used for signature evaluation and model development [8]. External evaluation used the GSE65858 microarray cohort described by Wichmann and colleagues [7].

Previously published breast cancer hypoxia and radiosensitivity signatures were first evaluated in TCGA HNSC [6]. We then developed a prognostic signature from hypoxia-related candidate genes assembled from published gene sets, using overall survival as the endpoint. Candidate genes were restricted to those represented in both expression datasets after gene annotation and alias resolution. GSE65858 was assessed for gene coverage and availability of survival data before model fitting; gene selection and coefficient estimation by LASSO Cox regression were performed in TCGA HNSC [9].

**Figure 1:**
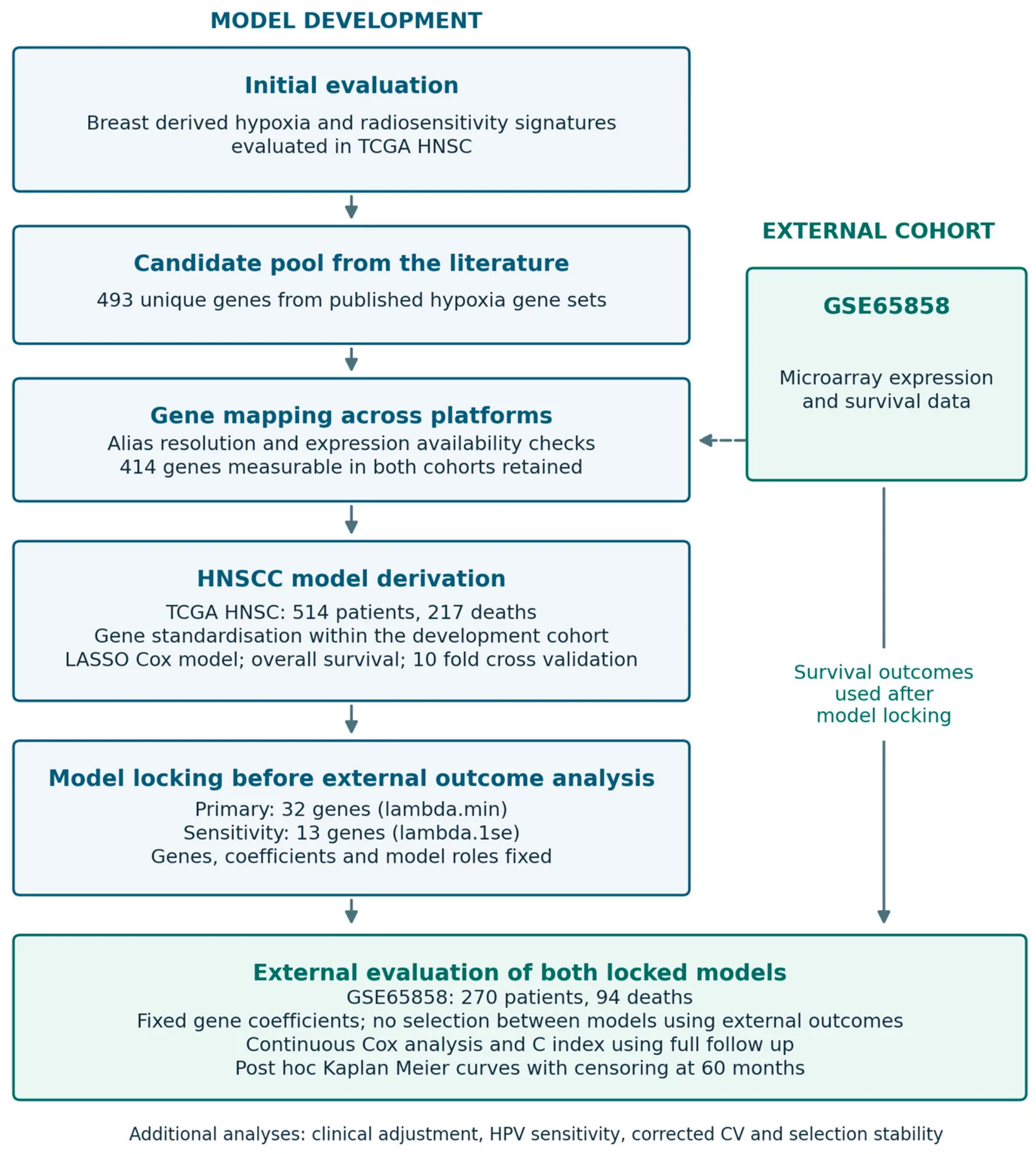
Study design and development of the HNSCC prognostic signatures derived from hypoxia-related genes. Breast-derived hypoxia and radiosensitivity signatures were first evaluated in TCGA HNSC. A literature informed pool of 493 unique hypoxia-related candidate genes was assembled. Gene annotation and alias resolution identified 414 genes measurable in both TCGA HNSC and GSE65858, which formed the frozen candidate pool. LASSO Cox models were derived using overall survival in TCGA HNSC (514 patients; 217 deaths), with 10-fold cross validation used to select the penalty. The 32-gene lambda.min model and the 13-gene lambda.1se model were assigned primary and sensitivity roles, respectively, and their genes and coefficients were locked before external outcome analysis. Both models were evaluated in GSE65858 (270 patients; 94 deaths) without coefficient refitting or model selection based on the external results. The dashed arrow denotes the use of GSE65858 expression availability during candidate filtering; its survival outcomes were used for subsequent external evaluation. Continuous Cox analyses and C indices used full follow-up. Kaplan–Meier analyses used a cohort median score split and administrative censoring at 60 months. Additional analyses included clinical adjustment and checks of development procedures. The 60-month Kaplan–Meier analysis was a post hoc secondary analysis.

After derivation, the lambda.min model was designated as the primary signature and the lambda.1se model as a sensitivity analysis, before their associations with survival were evaluated in GSE65858. The selected genes and their coefficients were fixed before external evaluation. Both models were applied to GSE65858 without refitting their gene coefficients, and both results were reported. The study workflow is shown in Figure 1.

### 2.2. TCGA HNSC Development Cohort

TCGA HNSC provided the tumour gene expression profiles and clinical annotations for model development [8]. Primary solid tumours and metastatic samples were identified by TCGA sample type codes 01 and 06, respectively [10]. The combined dataset contained 517 sample records. Excluding the two metastatic samples left 515 primary tumour records.

Overall survival time, recorded in months, and vital status were taken from the supplied clinical annotations. Death was coded as an event, and patients recorded as living were censored at their reported survival time. Records with missing or nonpositive survival times were excluded. One primary tumour record had missing survival time; its exclusion yielded 514 unique patients, with 217 deaths and 297 censored observations.

### 2.3. GSE65858 External Evaluation Cohort

The external evaluation used GSE65858, an HNSCC cohort available through the Gene Expression Omnibus and described by Wichmann and colleagues [7]. Tumour gene expression was measured using the Illumina HumanHT-12 v4.0 Expression BeadChip (GPL10558). The cohort included patients with tumours of the oral cavity, oropharynx, larynx and hypopharynx.

The analysis included 270 patients with gene expression data, overall survival time and event status. There were 94 deaths during the available follow-up. The 32-gene primary model and the 13-gene sensitivity model were evaluated in the same patients. Overall survival was the endpoint for both models, and the continuous Cox analyses used the full available follow-up.

### 2.4. Breast-Derived Hypoxia and Radiosensitivity Signatures

The previously derived breast cancer hypoxia signature comprised *CP*, *GPC3* and *STC1*. The DNA repair radiosensitivity (RS) signature comprised *ATR*, *RPA2*, *BLM* and *MRE11A*. Both signatures were developed in TCGA BRCA, with RS derivation restricted to patients who received radiotherapy [6]. The supplied gene coefficients were applied to TCGA HNSC without reestimation (Table 1).

For full-cohort scoring, expression values were transformed as log2(x + 1). Each gene was then standardised using its mean and sample standard deviation across the 515 primary tumour records. Scores were calculated as weighted sums of standardised expression using the coefficients below and were mean centred. Survival eligibility filters were applied after scoring, yielding the 514 patients included in the survival analyses.

For the RS analysis restricted to the 119 patients with recorded postoperative radiotherapy, genes were standardised again within that subset before calculating and centring the score. For the RS by radiotherapy interaction analysis, standardisation was performed jointly across the 180 patients with known radiotherapy status, using a shared mean and standard deviation for each gene across both treatment groups. The supplied positive RS coefficients were used directly, without a further sign inversion.

### 2.5. Published HNSCC Hypoxia Signatures

The Toustrup 15-gene panel was evaluated in TCGA HNSC as a continuous gene set score [2]. All 15 genes were included. After log2(x + 1) transformation and gene-wise standardisation, their expression values were averaged with equal weights, and the resulting score was mean centred. This scoring method was an adaptation of the published panel. The same 15-gene panel was also evaluated in GSE65858 by averaging its gene-wise z scores within that cohort.

The Eustace 26-gene panel was evaluated in GSE65858. The published assay uses quantitative PCR to calculate the median panel expression normalised to the geometric mean of three reference genes, *GNB2L1*, *B2M* and *RPL11* [3]. For the microarray analysis, 25 panel genes were available; *HILPDA*, also known as *HIG2*, was unavailable. Panel and reference genes were individually z-scored within GSE65858. For each patient, the score was calculated as the median of the 25 panel z scores minus the arithmetic mean of the three reference gene z scores. This procedure produced an adapted score and did not reproduce the original quantitative PCR assay.

No outcome-based gene selection or coefficient estimation was used to construct either comparator score. The comparisons in GSE65858 were undertaken after external evaluation of the two HNSCC models and were considered exploratory.

### 2.6. Construction of the Hypoxia Candidate Gene Pool

Candidate genes were assembled from eight source lists. The Hallmark hypoxia set and the Winter, Buffa, Elvidge and Fardin lists were obtained from MSigDB. These were combined with the Toustrup panel, the Eustace derived list used at the pool assembly stage and the three genes in the breast hypoxia score. The contributions recorded in the candidate file are shown in Table 2.

These counts describe the lists used for candidate assembly, which can differ from the sizes of the original published signatures. At this stage, the Eustace derived list excluded *ANGPTL4* and *KRT17*, which had not been identified in the supplied TCGA expression data. *ANGPTL4* was nevertheless represented in the combined pool through other source lists.

The union contained 493 unique gene symbols. Source membership and the number of contributing lists were retained for each gene. After annotation and alias resolution, 453 candidates were mapped in TCGA HNSC and 442 in GSE65858. The 414 genes represented in both datasets were retained, and this candidate pool was fixed before LASSO Cox model fitting.

### 2.7. Gene Annotation and Cross-Platform Harmonisation

Gene identifiers were reconciled between the TCGA HNSC expression data and the GSE65858 microarray data. GSE65858 was profiled on the Illumina HumanHT-12 v4.0 platform [7]. Probe identifiers were mapped to gene symbols using the supplied GPL10558 annotation file. For genes represented by multiple probes, expression was calculated for each patient as the arithmetic mean across all probes mapped to that gene. The same aggregation rule was applied throughout, without selecting probes according to their association with survival.

Candidate symbols were matched directly to the gene identifiers in each dataset. For unmatched candidates, older or alternative symbols were examined. The mapping record retained the candidate symbol, match type, matched alias and coverage in each cohort. Aliases were recorded separately for the two datasets because a gene could require an alternative symbol in one cohort but match directly in the other. For example, *MRGBP* matched directly in TCGA HNSC and through *C20orf20* in GSE65858.

Genes without a match in either dataset after this reconciliation were excluded from the development pool. The complete candidate mapping and the frozen list retained source membership and cohort-specific mapping details, allowing the 414-gene intersection to be traced to the original 493 candidates. A summary of candidate coverage and alias mapping is provided in Supplementary Table S3; the full candidate gene coverage and mapping record is provided in Data S1.

### 2.8. LASSO Cox Signature Derivation

The HNSCC signature was developed in TCGA HNSC using the frozen pool of 414 genes and overall survival as the endpoint. Expression values were transformed as log2(x + 1) and each gene was z-scored within the development cohort. A Cox proportional hazards model with a least absolute shrinkage and selection operator (LASSO) penalty was fitted [9]. The analysis used CoxnetSurvivalAnalysis from scikit-survival version 0.25.0 in Python version 3.12.3, with l1_ratio set to 1.0 to apply an L1 penalty [15].

Penalty strength was selected by 10-fold cross validation in TCGA HNSC, with a fixed random state. Genes were standardised once across all 514 development patients before fold assignment. The penalty grid was obtained from a model fitted to the full development data. The cross-validation output comprised 100 penalty values, with the mean concordance index (C index) and its standard error recorded at each value. Penalty strength is termed alpha in the software; the labels lambda.min and lambda.1se are used here for the two selected models. The lambda.min model used the penalty yielding the highest mean cross-validated C index. The lambda.1se model used the largest penalty whose mean C index was at least the best mean C index minus its standard error. In a subsequent verification check, cross validation was repeated with each gene mean and standard deviation estimated within the training fold and applied unchanged to the corresponding held out fold. This check retained the original fold assignments and penalty grid. The penalty grid had therefore still been obtained from the full development cohort. The check did not use external outcomes or change the locked signatures.

The resulting models contained 32 and 13 genes with nonzero coefficients, respectively. Gene lists and penalised coefficients were retained for both models. After derivation and before testing their survival associations in GSE65858, the 32-gene lambda.min model was designated as primary and the 13-gene lambda.1se model as a sensitivity analysis. Both sets of genes and coefficients were fixed for external evaluation. The locked genes and coefficients are provided in Supplementary Tables S1 and S2.

Gene selection stability was examined in a separate diagnostic analysis using 200 bootstrap samples of the 514 development patients. Each sample was drawn with replacement, gene expression was standardised within that sample, and LASSO Cox regression was fitted at fixed penalties of 0.039161227341523885 and 0.06532486436580959, matching the selected values at full precision. Selection frequency was the proportion of successful fits in which a gene had a nonzero coefficient. This analysis assessed stability conditional on fixed penalties, rather than variability from repeating penalty selection. It did not change the locked gene coefficients.

### 2.9. External Evaluation and Score Calculation

The two locked signatures were evaluated in GSE65858, the external cohort described by Wichmann and colleagues [7]. Following probe-to-gene mapping and averaging of multiple probes, each signature gene was standardised within GSE65858 by subtracting its cohort mean and dividing by its cohort standard deviation. For each patient, the signature score was calculated as the sum of these gene z scores multiplied by their corresponding penalised coefficients from TCGA HNSC. This calculation was performed separately for the 32-gene primary signature and the 13-gene sensitivity signature. Thus, gene coefficients were transferred from TCGA HNSC, whereas the expression means and standard deviations were estimated in GSE65858. Each composite score was mean-centred, without subsequent division by its standard deviation. The original hazard ratios represent a one-unit increase in the weighted composite score. We additionally report hazard ratios per standard deviation of each composite score. For these estimates, the existing score was divided by its sample standard deviation in the analysis population. Gene standardisation and the locked gene coefficients were unchanged when restricting an analysis to complete cases.

Both signatures were evaluated in the same 270 patients using overall survival over the full available follow-up. Associations between the continuous scores and survival were assessed by Cox regression, with hazard ratios, 95% confidence intervals and *p* values reported for each signature. Discrimination was summarised using the C index. The external Cox analyses evaluated the composite scores; the individual gene coefficients used to construct them were not reestimated. Both models were reported in their assigned roles, without selecting a preferred signature from the external results.

### 2.10. Clinical Adjustment in GSE65858

During revision, each locked signature was evaluated separately in an unpenalised Cox model adjusted for continuous age, UICC stage grouped as I or II, III, or IV, tumour site, HPV DNA status and recorded smoking status. Stage, site, HPV and smoking were entered as categorical variables. Reference categories were stage I or II, oropharynx, HPV-negative and no recorded smoking, respectively. The main model retained the three HPV DNA categories of negative, HPV16 and other HPV. Complete cases on these variables and survival data comprised 265 patients with 92 deaths. A clinical-factors-only model was also fitted to these 265 patients. The original scores from the 270-patient cohort were retained.

Sensitivity analyses used the 246 patients with 81 deaths who also had a detailed HPV16 DNA and RNA classification. Both HPV specifications were fitted on this same patient subset using unpenalised Cox regression. The simple DNA specification had two observed categories in this subset, negative and HPV16, because all patients classified as other HPV lacked the detailed classification. The detailed specification distinguished DNA-negative, DNA-positive and RNA-positive, and DNA-positive and RNA-negative tumours. Matching clinical models without a signature score were also fitted on this subset for comparison. C indices for these combined and clinical models describe apparent discrimination in the patients used to fit them. Proportional hazards diagnostics for models containing a score used Schoenfeld residuals.

### 2.11. Survival and Statistical Analyses

Overall survival associations were assessed using Cox proportional hazards regression in lifelines version 0.30.0, with signature scores entered as continuous variables [16]. For the breast-derived RS score in TCGA HNSC, the interaction analysis included the centred score, recorded postoperative radiotherapy status and their product term. Radiotherapy was coded as 1 for Yes and 0 for No, and the analysis was restricted to patients with known treatment status and complete model variables. The interaction term assessed whether the score survival association differed between the two recorded treatment groups.

Survival distributions were estimated using the Kaplan–Meier method [17]. For graphical comparisons, patients were divided into higher and lower score groups using the median score in the cohort being analysed, and the groups were compared using the log-rank test [18]. For the two locked HNSCC signatures, continuous Cox analyses were the main association analyses; median-split curves and log-rank tests provided supplementary descriptions.

The GSE65858 Kaplan–Meier analyses were repeated with administrative censoring at 60 months after the initial external evaluation. This was a post hoc secondary analysis intended to limit the sparse late follow-up tail. Observed survival times were replaced by the lesser of the recorded time and 60 months. Deaths at or before 60 months remained events; deaths after 60 months were recoded as censored at 60 months. Patients already censored at or before 60 months retained their original follow-up times. Log-rank tests were repeated using these data. Continuous Cox analyses and their C indices retained full follow-up. Censoring marks and numbers at risk were added to the primary Kaplan–Meier plot.

### 2.12. Adjusted and Exploratory Analyses in TCGA HNSC

In TCGA HNSC, the breast-derived hypoxia and RS scores and the adapted Toustrup score were each evaluated in Cox models adjusted for age, T stage, N stage and smoking history. These adjusted analyses did not include the newly derived HNSCC signatures. Age was entered as a continuous variable. T stage was grouped as T1 to 2 or T3 to 4, and N stage as N0 or node-positive. Smoking history was coded as never or ever smoking. Patients with missing age or unclassified stage or smoking covariates were excluded from these models. An L2 penalty of 0.001 was applied to the Cox regression coefficients. The gene weights were unchanged, and the scores calculated in the full cohort were retained when restricting these adjusted analyses to eligible patients.

Separate sensitivity analyses added recorded p16 status or radiotherapy status to the same clinical covariates and included patients with complete data for the relevant model. Further exploratory univariable analyses evaluated each score separately in the p16-positive and p16-negative groups. These subgroup analyses were undertaken after review of the initial results.

The treatment interaction, p16 subgroup, adapted signature comparisons and additional analyses undertaken during revision were exploratory. No adjustment for multiple testing was applied. Nominal *p* values are reported alongside effect estimates and confidence intervals; isolated significant results in small subsets were not treated as confirmatory evidence.

## 3. Results

### 3.1. Cohort Characteristics

The TCGA HNSC development cohort comprised 514 patients, with 217 deaths (42.2%) during the available follow-up. Median age at diagnosis was 61 years, and 381 patients (74.1%) were male and 133 (25.9%) were female. Age and sex were recorded for all included patients. Postoperative radiotherapy was recorded as received in 119 patients (23.2%) and not received in 61 (11.9%); this field was missing for 334 patients (65.0%). Consequently, the RS analysis in treated patients included 119 patients, while the treatment interaction analysis included the 180 patients with known radiotherapy status. Cohort characteristics are compared in Table 3.

The GSE65858 external evaluation cohort comprised 270 patients, with 94 deaths (34.8%) over the full available follow-up. Tumour subsite was recorded for 266 patients: 102 had oropharyngeal tumours, 83 oral cavity tumours, 48 laryngeal tumours and 33 hypopharyngeal tumours. Subsite was missing for four patients. HPV DNA status was available for 269 patients, comprising 196 negative, 60 HPV16 and 13 other HPV results; one patient had no recorded result. Both locked HNSCC signatures were evaluated in all 270 patients. There were 223 male and 47 female patients. Age, UICC stage and smoking status were recorded for all 270 patients. The 265 patients eligible for the main adjusted analysis included 92 deaths; the subset with detailed HPV information included 246 patients and 81 deaths.

### 3.2. Breast-Derived Signatures in TCGA HNSC

In the full TCGA HNSC cohort, the breast-derived hypoxia score had a hazard ratio of 0.81 for overall survival (95% CI 0.53 to 1.23; *p* = 0.32; 514 patients, 217 deaths). For the RS score, the main analysis among patients with recorded postoperative radiotherapy yielded an HR of 0.59 (95% CI 0.29 to 1.22; *p* = 0.16; 119 patients, 44 deaths). The secondary RS analysis in the full cohort yielded an HR of 0.79 (95% CI 0.59 to 1.06; *p* = 0.11). These hazard ratios represent a one-unit increase in the respective weighted score. All three estimates were below 1, but their confidence intervals included 1. Full-cohort median-split curves for the breast-derived hypoxia and RS scores are shown in Figures S2 and S3, respectively.

Among the 180 patients with known postoperative radiotherapy status, including 65 deaths, the RS score by radiotherapy interaction had an exponentiated coefficient of 0.73 (95% CI 0.20 to 2.65; *p* = 0.63). This analysis did not provide clear evidence that the RS score survival association differed by recorded radiotherapy status. The wide interval leaves substantial uncertainty about the magnitude and direction of any interaction.

### 3.3. Adjusted and Exploratory Breast Signature Analyses

The main adjusted analyses included 404 patients and 161 deaths. After adjustment for age, grouped T and N stage, and smoking history, the HR was 0.77 for the hypoxia score (95% CI 0.46 to 1.31; *p* = 0.33) and 0.86 for the radiosensitivity score (95% CI 0.58 to 1.26; *p* = 0.43).

The sensitivity analysis that additionally included p16 status comprised 82 patients and 22 deaths. The adjusted HR was 0.23 for the hypoxia score (95% CI 0.05 to 0.94; *p* = 0.04) and 0.40 for the radiosensitivity score (95% CI 0.13 to 1.29; *p* = 0.13). In the separate analysis incorporating radiotherapy status, 152 patients and 54 deaths were included. The adjusted HR was 0.53 for the hypoxia score (95% CI 0.19 to 1.50; *p* = 0.23) and 0.43 for the radiosensitivity score (95% CI 0.21 to 0.89; *p* = 0.02). These estimates arose from different patient subsets and should be interpreted alongside the main adjusted analyses.

In the exploratory p16 stratified analyses, the positive group included 38 patients and four deaths. The HRs were 0.27 for the hypoxia score (95% CI 0.01 to 6.36; *p* = 0.41) and 0.62 for the radiosensitivity score (95% CI 0.09 to 4.31; *p* = 0.63). Among 71 p16-negative patients with 24 deaths, the corresponding HRs were 0.49 (95% CI 0.14 to 1.73; *p* = 0.27) and 0.70 (95% CI 0.26 to 1.84; *p* = 0.47). The wide confidence intervals, particularly in the p16-positive group, leave substantial uncertainty about the associations within these subgroups.

### 3.4. Published Hypoxia Signature Comparisons

In TCGA HNSC, the adapted score based on the Toustrup 15-gene panel [2] was associated with higher mortality in the univariable analysis of 514 patients with 217 deaths (HR 1.35, 95% CI 1.06 to 1.72; *p* = 0.02). After adjustment for age, grouped T and N stage, and smoking history, the HR was 1.30 (95% CI 0.99 to 1.72; *p* = 0.06; 404 patients, 161 deaths). The median-split analysis using full follow-up yielded a log-rank *p* value of 0.185.

In GSE65858, the adapted Toustrup score had an HR of 1.27 (95% CI 0.90 to 1.79; *p* = 0.179; C index 0.564; 270 patients, 94 deaths). Its confidence interval included 1, in contrast to the univariable result in TCGA HNSC.

In GSE65858, the adapted Eustace score [3], calculated from 25 available panel genes and three reference genes, showed little evidence of an association with overall survival among 270 patients with 94 deaths (HR 0.99, 95% CI 0.79 to 1.25; *p* = 0.94). The C index was 0.499 when oriented by the fitted Cox coefficient. Median-split log-rank *p* values were 0.69 with full follow-up and 0.84 after administrative censoring at 60 months. This exploratory comparison concerns the microarray adaptation described in the Methods, rather than the original quantitative PCR assay.

### 3.5. Candidate Gene Pool and Cross-Cohort Coverage

Combining the eight source lists produced 493 unique candidate genes. After the documented alias resolution, 453 genes were identified in TCGA HNSC and 442 in GSE65858. The intersection comprised 414 genes, representing 84.0% of the candidate pool, and these genes formed the frozen set used for model development. Twelve genes required an alias match in at least one cohort. The cohort-specific totals included 442 direct and 11 alias matches in TCGA HNSC, and 433 direct and nine alias matches in GSE65858.

### 3.6. Derivation of the HNSCC Signatures

LASSO Cox regression was applied to the 414-gene pool in the TCGA HNSC development cohort of 514 patients with 217 deaths. The penalty with the highest mean C index across the 10 cross-validation folds, designated lambda.min, was 0.03916. Its mean C index was 0.628 (standard error 0.019), and the fitted model contained 32 genes with nonzero coefficients. Applying the one standard error rule selected a penalty of 0.06532 and a 13-gene model, designated lambda.1se. At this penalty, the mean cross-validation C index was 0.609 (standard error 0.019).

The 32-gene model was designated as the primary signature and the 13-gene model as the sensitivity model before their external survival associations were evaluated. Both sets of genes and coefficients were fixed for application to GSE65858. The cross-validation estimates were used to select the penalties and therefore represent model selection performance within the development cohort.

In the subsequent cross-validation check using standardisation within training folds, the mean C index was 0.6279 (standard error 0.0194) at the primary penalty and 0.6098 (standard error 0.0198) at the sensitivity penalty. The selected penalties and the final sets of 32 and 13 genes were unchanged, and the fitted gene coefficients matched the locked models. The primary mean C index was 0.6277 in the original analysis. This check did not show an appreciable change in the primary estimate, although the use of a penalty grid derived from the full cohort remained a limitation.

In the bootstrap stability check, the saved outputs report 200 successful fits at each exact fixed penalty. The repeat run captured warnings during every fit; all 200 fits at each penalty completed without a fitting warning. Among the locked genes, 13 of 32 primary genes and five of 13 sensitivity genes were selected in at least half of resamples; three and one, respectively, were selected in at least 80%. *PGK1* was selected in 91.0% and 84.5% of resamples, and *HK1* in 82.5% and 69.5%, respectively. Mean selection frequencies across locked genes were 49.3% and 47.0%. These findings indicate variable selection stability beyond the most frequently retained genes.

### 3.7. External Evaluation in GSE65858

Both fixed signatures were evaluated in 270 patients with 94 deaths. In the continuous Cox analysis using full follow-up, a higher primary 32-gene score was associated with higher mortality (HR 1.81, 95% CI 1.12 to 2.95; *p* = 0.016). The corresponding C index was 0.566. The 13-gene sensitivity score was also associated with higher mortality (HR 2.65, 95% CI 1.13 to 6.19; *p* = 0.024), with a C index of 0.581. These analyses used the fixed genes and coefficients from TCGA HNSC, without refitting the signatures in GSE65858. The composite score standard deviations in GSE65858 were 0.4281 and 0.2543, respectively. Expressed per score standard deviation, the univariable HRs were 1.290 (95% CI 1.048 to 1.588) and 1.281 (95% CI 1.033 to 1.590), with unchanged *p* values. The larger original HR per score unit for the 13-gene model should therefore not be interpreted as a larger effect on a common scale.

**Figure 2:**
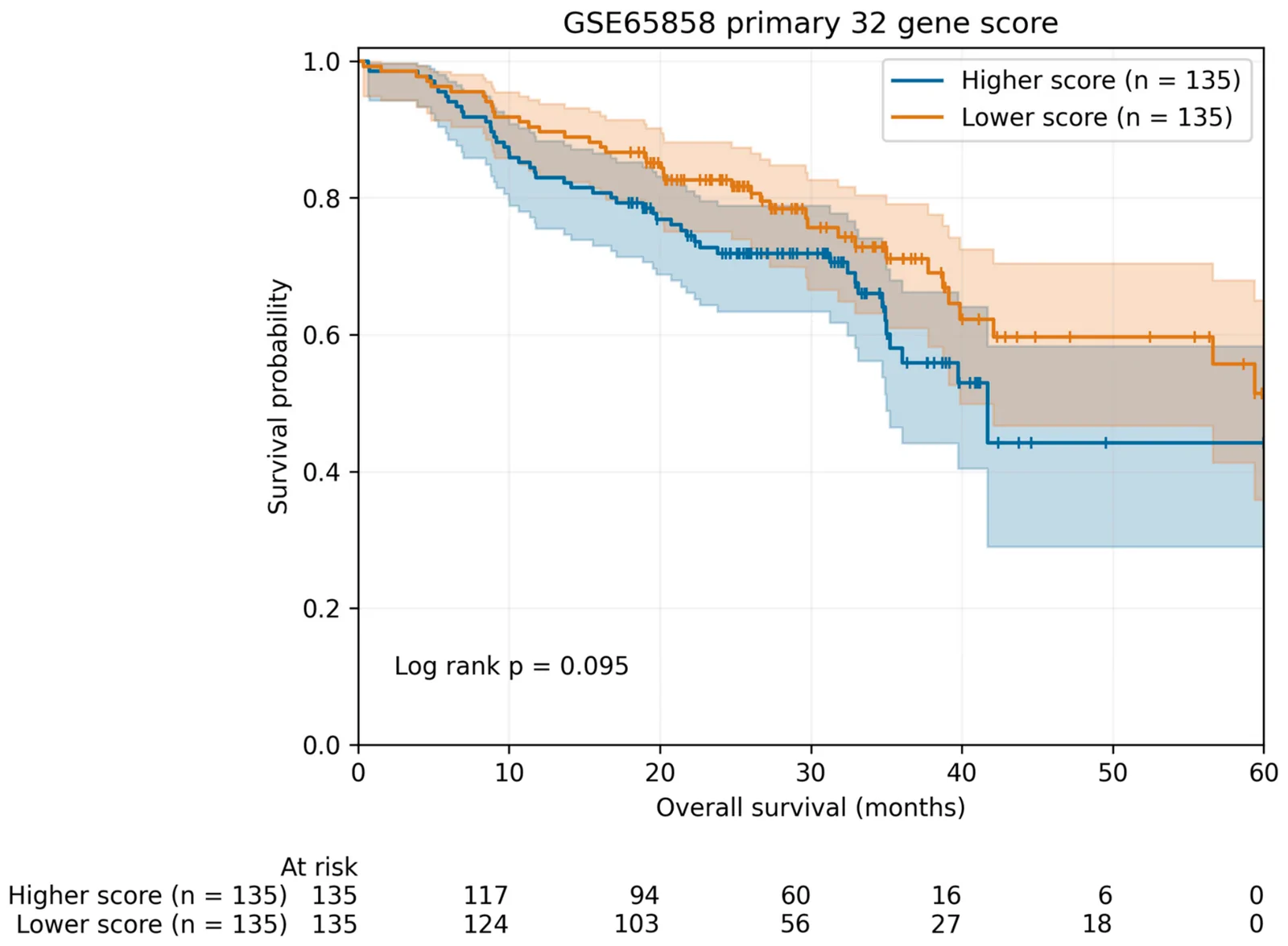
Overall survival in GSE65858 according to the primary 32-gene HNSCC score. Patients were divided at the cohort median into higher (score at or above the median; n = 135) and lower (n = 135) score groups. Follow-up was administratively censored at 60 months, retaining 88 deaths. Shaded bands show 95% confidence intervals and tick marks indicate censoring. Numbers at risk are shown at the end of each indicated time; the zero counts at 60 months reflect administrative censoring of all remaining observations. The log-rank *p* value was 0.095. This post hoc median-split analysis is secondary to the continuous Cox analysis with full follow-up, which gave an unadjusted HR of 1.29 per score standard deviation (95% CI 1.05 to 1.59; *p* = 0.016; C index 0.566). The adjusted continuous score results are presented separately in Table 4.

For the five-year Kaplan–Meier analyses, 16 patients had recorded follow-up beyond 60 months, including six whose deaths occurred after that point. Censoring these patients at 60 months reduced the number of deaths included in the analysis from 94 to 88. After division at each score’s cohort median, the log-rank *p* value was 0.095 for the primary signature (Figure 2) and 0.204 for the sensitivity signature (Figure S1). These restricted follow-up, median-split analyses were separate from the continuous Cox analyses above, which retained full follow-up.

### 3.8. Clinical Adjustment and HPV Sensitivity Analyses

In the main adjusted analysis of 265 patients with 92 deaths, the primary signature had an HR of 1.235 per score standard deviation (95% CI 0.987 to 1.546; *p* = 0.065). The corresponding HR for the 13-gene signature was 1.202 (95% CI 0.955 to 1.512; *p* = 0.117). The clinical-factors-only model had an apparent C index of 0.7010. Adding the primary or sensitivity score gave 0.7049 or 0.7046, increases of 0.0039 or 0.0036 within the same 265 patients. Neither association reached statistical significance after adjustment for age, grouped UICC stage, site, HPV DNA status and smoking. These models included several clinical factors simultaneously and do not isolate the contribution of HPV to the attenuation.

In the common subset of 246 patients, adjusted HRs remained nonsignificant under both HPV specifications (Table 4). On this subset, apparent C indices for the simple HPV clinical model and the models including the primary or sensitivity score were 0.70055, 0.70188 and 0.70165, respectively. With detailed HPV classification, the corresponding values were 0.70387, 0.70520 and 0.70394. Increments relative to the matching clinical model ranged from 0.00007 to 0.00133. These descriptive comparisons did not demonstrate a substantial improvement in apparent discrimination. Proportional hazards diagnostics detected no evidence of violation in the six adjusted models containing a score across the main and subset analyses.

Each model included one locked signature plus age, grouped UICC stage, tumour site, HPV status and smoking. HRs are per sample standard deviation of the existing composite score in the population analysed. The primary adjustment uses 265 patients; a matching clinical-factors-only model gave an apparent C index of 0.7010. Both HPV specifications were additionally compared on the same 246 patients; this subset contained no other HPV category. Gene coefficients remained fixed.

## 4. Discussion

This study developed two prognostic signatures from hypoxia-related genes in TCGA HNSC and found unadjusted associations with overall survival when their fixed coefficients were applied to GSE65858. However, neither signature retained statistical significance after clinical adjustment, and external discrimination of the scores alone was modest, with C indices of 0.566 and 0.581. The 265-patient main analysis and the 246-patient HPV sensitivity analyses showed only small increases in apparent discrimination when a score was added to the corresponding clinical factors model. The evidence therefore does not establish independent prognostic value or justify individual treatment decisions. Defining the model roles before external survival testing and reporting both models limited selection based on the external results, but these design features do not overcome the limitations of their observed performance.

The present workflow combines published hypoxia gene lists with existing tumour expression and survival data, allowing candidate signatures to be developed and evaluated without generating new experimental data. This provides a practical basis for prioritising models for further study. It does not replace validation against an independent measure of hypoxia or evaluation in cohorts with adequate treatment annotation.

The breast-cancer derived signatures produced less consistent evidence of association in TCGA HNSC. Their confidence intervals included 1 in the main univariable and adjusted analyses, although the hypoxia score in the p16-adjusted subset and the RS score in the radiotherapy-adjusted subset had nominal *p* values below 0.05. These subset findings qualify any simple conclusion that the breast signatures failed to transfer. They arose in smaller, differently selected patient groups and do not by themselves establish a reproducible protective association. Likewise, the imprecise RS by radiotherapy interaction estimate does not exclude a difference in score associations by treatment status. Taken together, these results favour a cautious interpretation: the HNSCC-derived models showed unadjusted external prognostic associations, while the behaviour of the breast-derived scores in HNSCC remained uncertain.

The published signature comparisons require attention to how each score was constructed. The original Toustrup study developed a classifier for hypoxic modification of radiotherapy, whereas our TCGA HNSC analysis tested the mean standardised expression of its 15 genes as a continuous prognostic score [2]. The observed univariable association therefore concerns this adaptation and does not reproduce the original treatment response evaluation. Similarly, the Eustace assay used a 26-gene panel measured by quantitative PCR with reference gene normalization [3]. Our GSE65858 implementation used 25 available genes and combined gene-wise z scores from microarray data. Its near-null survival association cannot establish that the original assay lacks value, because the assay, scoring procedure and clinical question differ.

The adapted Toustrup and Eustace scores were evaluated alongside the new HNSCC signatures in GSE65858. Their different *p* values do not establish a statistically significant difference in performance; the C indices for the primary HNSCC model and adapted Toustrup score were similar, at 0.566 and 0.564, respectively. These comparisons provide context for further benchmarking, without establishing superiority over published signatures. More broadly, selecting genes from hypoxia-related lists and fitting their weights against survival does not by itself show that the resulting score measures tumour oxygenation. The present evidence supports unadjusted prognostic associations, with uncertain independence from clinical factors; a biological interpretation as a quantitative hypoxia measure would require separate validation.

HPV-positive and HPV-negative HNSCC differ in prognosis and may differ in how a hypoxia-related score is distributed. The NIMRAD trial included both groups and found a lower median 26-gene hypoxia score in HPV-positive than in HPV-negative cancers [19]. Combining these groups could complicate the interpretation of a single score or cutoff across HNSCC, but their combination cannot be identified as the reason NIMRAD did not show treatment benefit: the trial also found no clear nimorazole benefit in its HPV-negative subgroup [19]. NIMRAD tested benefit from a hypoxia modifier, whereas our analyses concern overall survival associations. Our data do not test whether HPV status changes treatment benefit or the effect of either new score. Separate signature development by p16 status was not undertaken because p16 results were available for only 109 of the 514 development patients, including just four deaths in the p16-positive group.

Several limitations affect interpretation. The analyses were retrospective, and external evaluation was limited to one cohort. Clinical adjustment did not establish an independent association for either signature. The complete case analyses may differ from the full external cohort, and the detailed HPV subset excluded all patients classified as other HPV. HPV DNA status, HPV16 DNA and RNA classification, and p16 immunohistochemistry are different measurements and should not be treated as interchangeable. In TCGA HNSC, p16 status was missing in 405 of 514 patients and postoperative radiotherapy status in 334. The treatment and p16 analyses therefore concerned selected subsets. Only four deaths occurred in the p16-positive group, leaving very imprecise estimates. These analyses were exploratory, with no adjustment for multiple testing.

The external evaluation transferred fixed gene coefficients, but it did not transfer a fully fixed scoring procedure. Gene means and standard deviations were estimated within GSE65858, making each score dependent on the expression distribution of that cohort. The present implementation therefore does not provide a validated way to score an isolated future patient. Clinical application would require a defined assay, a fixed reference for expression standardisation, and prospective evaluation of the complete procedure, including its performance in formalin-fixed paraffin-embedded samples. Directly substituting RNA sequencing reference parameters into microarray data would not by itself resolve platform differences. GSE65858 also informed candidate gene availability before model development, although its outcomes were not used to fit the locked gene coefficients or choose between the models after evaluation.

Development cross validation was used to select penalty strength and is not an independent estimate of final model performance. The original analysis standardised genes before fold assignment. Repeating standardisation within each training fold produced similar mean C indices and left the selected penalties, genes and coefficients unchanged. However, the penalty grid still came from the full development cohort, and performance was not estimated through a separate nested validation procedure. Bootstrap selection frequencies also showed that many genes were not consistently retained across resamples; the check used fixed penalties and did not capture uncertainty from repeating penalty selection. Further studies should evaluate the fixed signatures alongside clinical models in additional cohorts and assess calibration if absolute survival probabilities are to be reported. Independent measurement of hypoxia would be needed to support a biological interpretation, while treatment selection would require evidence about treatment benefit.

## 5. Conclusions

Two prognostic signatures derived from hypoxia-related genes showed unadjusted associations with overall survival in GSE65858, but discrimination was modest and neither retained statistical significance after clinical adjustment. Their independent prognostic value remains unestablished. The findings support transparent evaluation of these exploratory candidates in additional cohorts, rather than clinical use. Neither a quantitative measure of tumour hypoxia nor prediction of treatment benefit has been demonstrated.

## Figures and Tables

**Table 1:** Coefficients used for the breast-cancer derived signatures.

Signature	Gene	Coefficient
Hypoxia	*CP*	0.27180250
Hypoxia	*GPC3*	0.09274532
Hypoxia	*STC1*	0.01305204
RS	*ATR*	0.004630324
RS	*RPA2*	0.334318088
RS	*BLM*	0.224064815
RS	*MRE11A*	0.004784271

**Table 2:** Sources of candidate gene pool.

Source List	Genes Before Combining Lists	Reference
Hallmark hypoxia	200	[11]
Winter	243	[1]
Buffa	50	[12]
Elvidge	172	[13]
Fardin	32	[14]
Toustrup	15	[2]
Eustace derived list	24	[3]
Breast hypoxia score	3	[6]

**Table 3:** Characteristics of the TCGA HNSC development cohort and the GSE65858 external evaluation cohort.

Characteristic	TCGA HNSC (N = 514)	GSE65858 (N = 270)
Patients	514	270
Age, mean (SD), years	60.9 (11.8)	60.1 (10.3)
Sex	Male 381; female 133	Male 223; female 47
Clinical stage	I 20; II 97; III 103; IVA 263; IVB 10; IVC 7; missing 14	I 18; II 37; III 37; IVA 155; IVB 16; IVC 7; missing 0
Primary tumour site	Oral tongue 128; larynx 116; oral cavity 70; floor of mouth 62; tonsil 43; base of tongue 26; buccal mucosa 22; alveolar ridge 18; hypopharynx 10; oropharynx 9; hard palate 7; lip 3; missing 0	Oropharynx 102; oral cavity (Cavum Oris) 83; larynx 48; hypopharynx 33; missing 4
HPV status	p16 positive 38; negative 71; missing 405	HPV DNA: negative 196; HPV16 60; other HPV 13; missing 1
Smoking history	Current 174; former 15 years or less 138; former more than 15 years 72; former duration unspecified 2; lifelong nonsmoker 116; blank 11; coded unknown 1	Yes 222; no 48; missing 0
Treatment field	Postoperative radiotherapy: yes 119; no 61; missing 334	Recorded modality: multi 189; mono 78; palliative 3; missing 0
Deaths	217 (42.2%)	94 (34.8%)
Median follow-up by reverse Kaplan–Meier, months	34.7	32.9

The cohorts used different stage, site, HPV and treatment fields. TCGA HPV was measured by p16 immunohistochemistry; GSE65858 HPV categories refer to DNA status. GSE65858 treatment modality does not establish radiotherapy receipt. Follow-up was estimated by reversing the overall survival event indicator.

**Table 4:** Clinically adjusted associations with overall survival in GSE65858.

Analysis	Patients/Deaths	Signature	HR per SD (95% CI)	*p* Value
Main, HPV DNA	265/92	32 genes	1.235 (0.987 to 1.546)	0.065
Main, HPV DNA	265/92	13 genes	1.202 (0.955 to 1.512)	0.117
Common subset, HPV DNA	246/81	32 genes	1.137 (0.898 to 1.439)	0.285
Common subset, HPV DNA	246/81	13 genes	1.113 (0.878 to 1.412)	0.376
Common subset, HPV DNA and RNA	246/81	32 genes	1.134 (0.896 to 1.435)	0.297
Common subset, HPV DNA and RNA	246/81	13 genes	1.100 (0.867 to 1.397)	0.432

## Data Availability

TCGA HNSC data are available through the Genomic Data Commons and FireBrowse, and GSE65858 is available through the Gene Expression Omnibus. Analysis code, locked signature coefficients, patient scores and supporting clinical annotations are available in the project repository (https://github.com/JimmyCarterOsei/TCGA-HNSC-hypoxia-radiosensitivity, accessed on 30 September 2026). Additional processed input data are available upon reasonable request to the corresponding author.

## Supplementary Materials

The following supporting information can be downloaded at: https://www.mdpi.com/article/10.3390/genes17101256/s1, Figure S1, Overall survival in GSE65858 according to the 13-gene sensitivity score; Figure S2, Overall survival in TCGA HNSC according to the breast-derived hypoxia score; Figure S3, Overall survival in the full TCGA HNSC cohort according to the breast-derived RS score; Tables S1 and S2, Locked signature genes and coefficients; Table S3, Candidate gene coverage summary and alias mapping; Data S1, Full candidate gene coverage and mapping (CSV).
