# Supplementary material for: Development and External Evaluation of Hypoxia-Related Prognostic Signatures in Head and Neck Squamous Cell Carcinoma
Source: Genes (Basel). 2026 Oct 11;17(10):1256. doi: 10.3390/genes17101256 (PMC13704498; doi:10.3390/genes17101256)
Supplement: Supplementary file 1 [file genes-17-01256-s001.zip › genes-4604915-supplementary.pdf]

# Supplementary figures

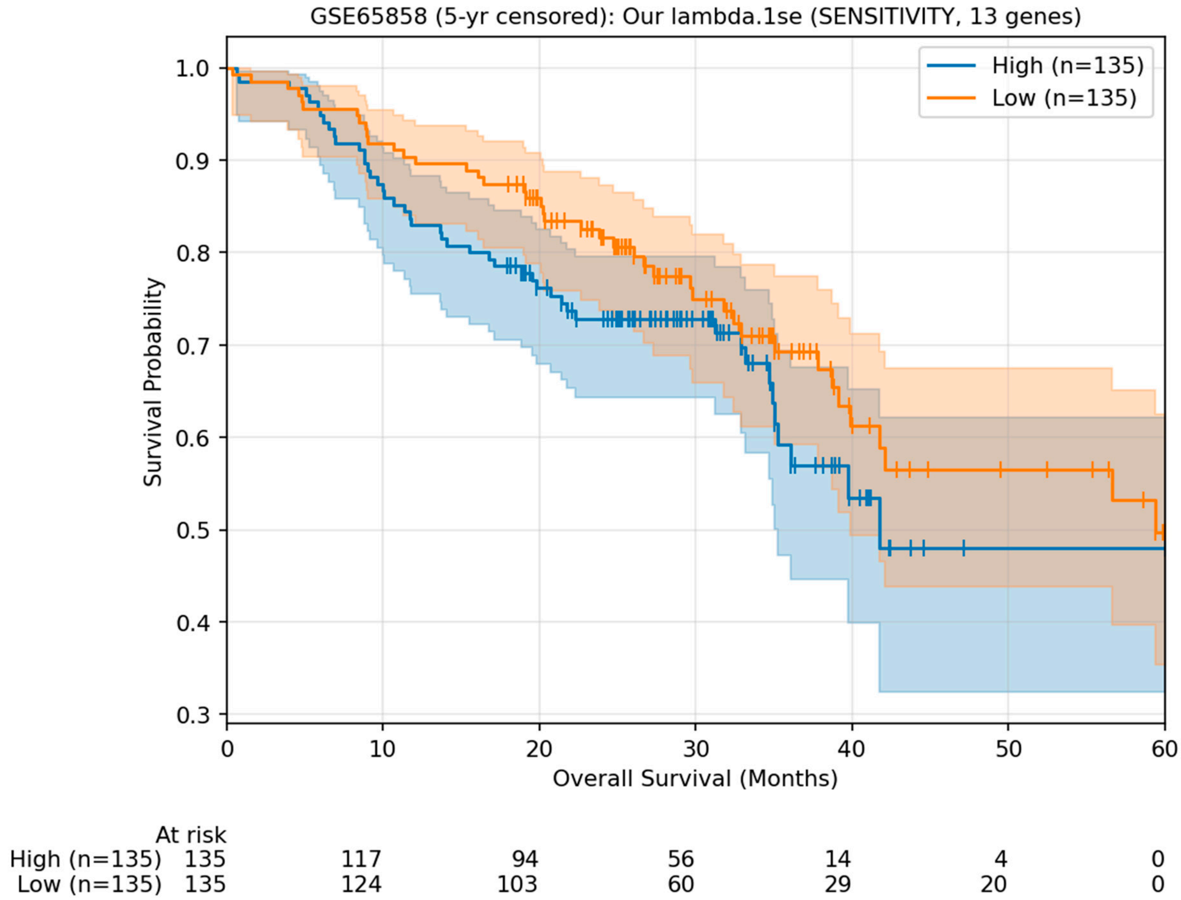


Figure S1. Overall survival in GSE65858 according to the 13 gene sensitivity score. The 270 patients were divided at the cohort median into higher (score at or above the median; n = 135) and lower (n = 135) score groups. Follow up was administratively censored at 60 months, retaining 88 deaths; shaded bands show 95% confidence intervals. Censoring ticks and numbers at risk are shown. The log rank p value was 0.204. The separate continuous Cox analysis used full follow up (94 deaths): HR 2.65 per score unit (95% CI 1.13–6.19; p = 0.024; C index 0.581). This HR does not describe the median split comparison.

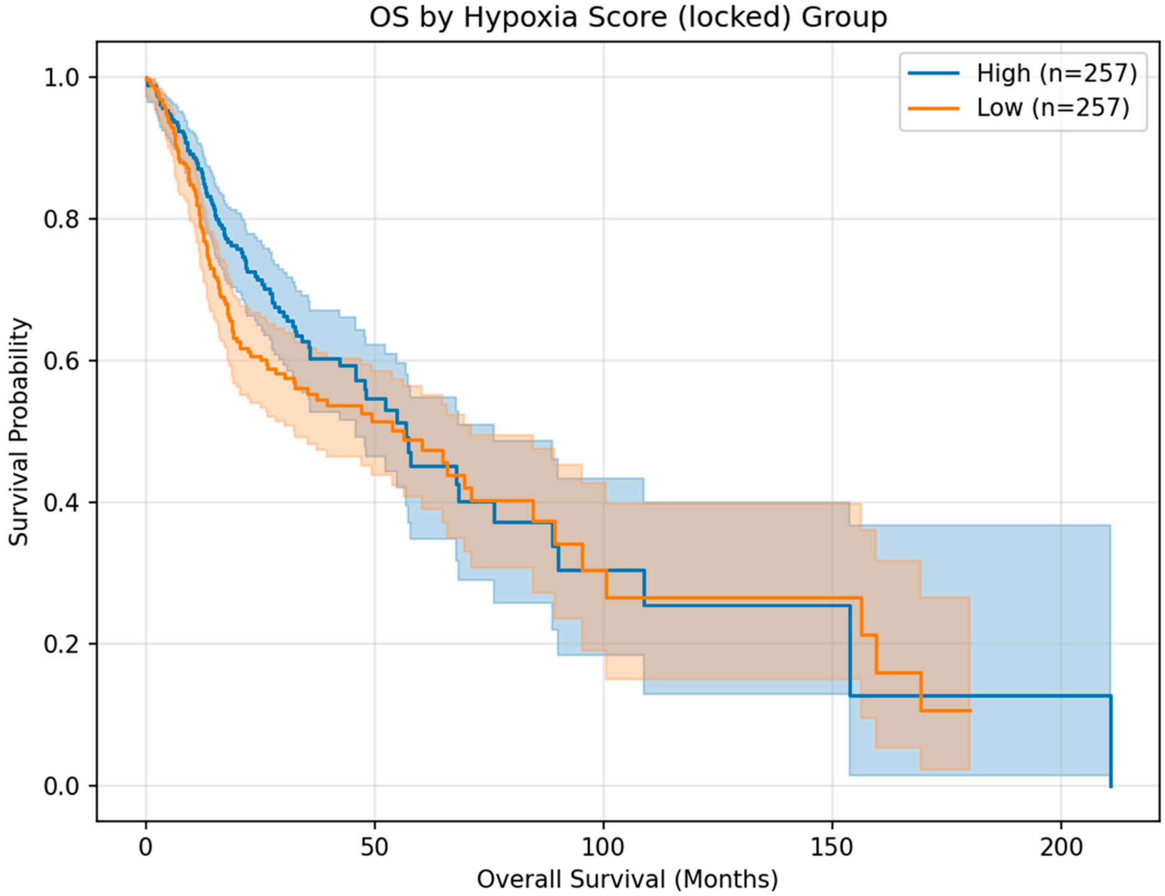


Figure S2. Overall survival in TCGA HNSC according to the breast derived hypoxia score. The analysis included 514 patients and 217 deaths over the full recorded follow up. Patients were divided at the cohort median into higher (score at or above the median; n = 257) and lower (n = 257) score groups; shaded bands show confidence intervals. The log rank p value was 0.14. The continuous Cox analysis gave an HR of 0.81 per score unit (95% CI 0.53–1.23; p = 0.32). The curve uses the fixed breast derived coefficients.

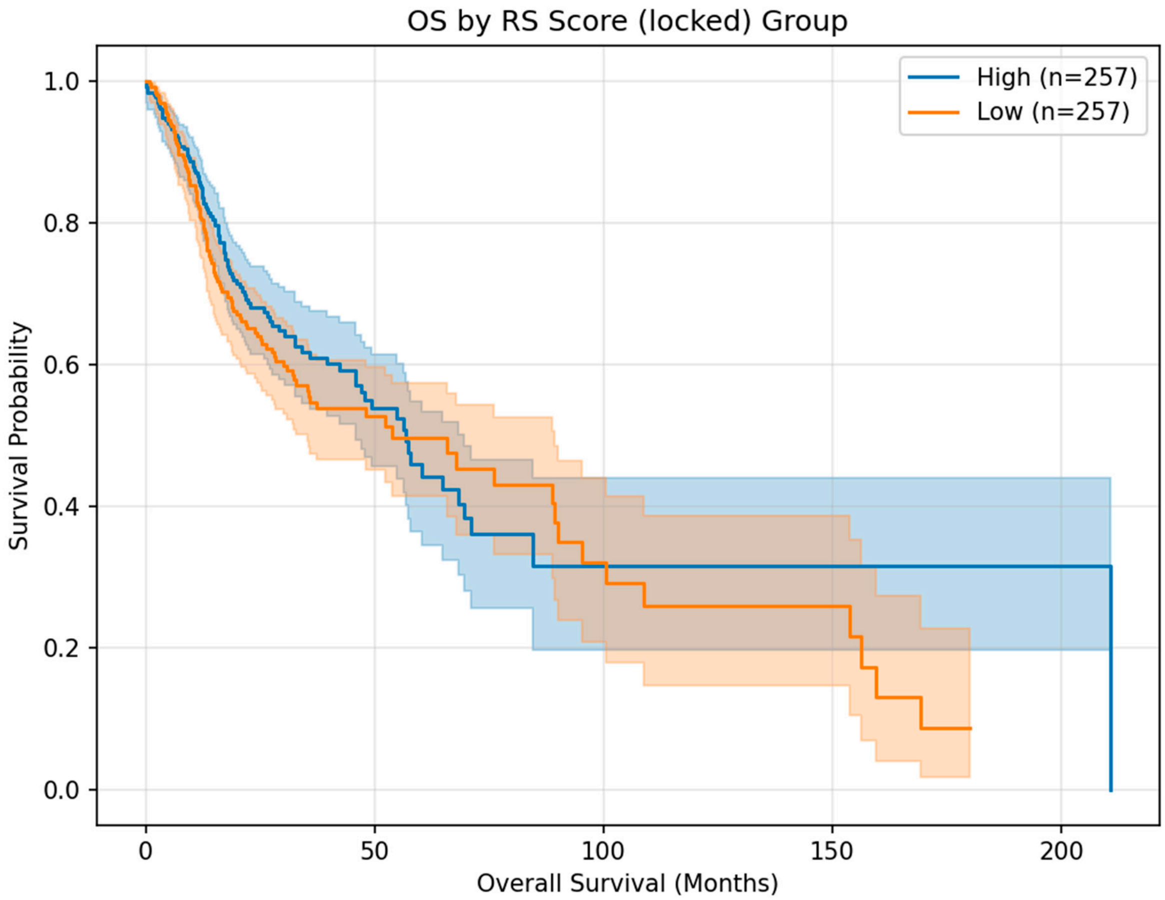


Figure S3. Overall survival in the full TCGA HNSC cohort according to the breast derived RS score. This was the secondary full cohort analysis (514 patients; 217 deaths), rather than the main RS analysis restricted to 119 patients with recorded postoperative radiotherapy. Patients were divided at the cohort median into higher (score at or above the median; n = 257) and lower (n = 257) score groups; shaded bands show confidence intervals. Full follow-up was retained. The log rank p value was 0.43; the separate continuous Cox analysis gave an HR of 0.79 per score unit (95% CI 0.59–1.06; p = 0.11).

## Supplementary tables

Table S1. Locked 32 gene primary signature and signed coefficients.

| **Gene** | **Coefficient** |
|---|---|
| SLC2A3 | 0.1452364484036017 |
| PGK1 | 0.1310610429752051 |
| MCTS1 | 0.1306819912414665 |
| HK1 | 0.109524800373964 |
| S100A4 | -0.104958043321486 |
| PKLR | -0.0966952144177822 |

| Gene | Coefficient |
|---|---|
| CSGALNACT1 | -0.07819886314287459 |
| KRT18 | 0.06494166377102779 |
| HS3ST1 | 0.0646166120492164 |
| ABCB1 | -0.064539775222585 |
| SPOCK1 | 0.060983876042131 |
| KIF5A | 0.0516765407094385 |
| SRPX | 0.0490021175231839 |
| CYB5A | 0.0476955388892332 |
| PLAU | 0.0457191247728376 |
| ANXA5 | 0.044503287401952 |
| SLC6A6 | -0.0441508432288808 |
| CCT6A | 0.0383085313178999 |
| EPO | -0.034276508688768 |
| PYGM | 0.0342181683209625 |
| SGSM2 | -0.0322901318141379 |
| AMPD3 | -0.0249063583690308 |
| EGF | 0.0231926298951571 |
| PTGS2 | -0.0225287741336515 |
| DDR1 | 0.0197801898173981 |
| BTG1 | -0.0163767061912812 |
| CD99 | -0.0071519755459105 |
| DTNA | 0.0047199726032973 |
| CHCHD2 | 0.0045174835651602 |
| MPI | 0.0019528234974323 |
| TGM2 | 0.0007562233286218 |
| INSIG2 | 2.186161639009012e-05 |

Coefficients multiply gene expression z scores. The genes and coefficients were fixed in TCGA HNSC before overall survival outcomes were evaluated in GSE65858.

Table S2. Locked 13 gene sensitivity signature and signed coefficients.

| Gene | Coefficient |
|---|---|
| PGK1 | 0.1180444697919895 |
| HK1 | 0.0667634764174852 |
| ABCB1 | -0.06477518007016279 |
| SPOCK1 | 0.059418483009155 |
| MCTS1 | 0.0496199298880562 |
| PKLR | -0.0451971882370234 |
| SLC2A3 | 0.0436549617781268 |
| KRT18 | 0.027995141105754 |
| PLAU | 0.0198652716329187 |
| SRPX | 0.0191699115362677 |
| S100A4 | -0.0162109453843623 |
| STC2 | 0.0159915948499156 |
| SGSM2 | -0.0054771730286082 |

Coefficients multiply gene expression z scores. The genes and coefficients were fixed in TCGA HNSC before overall survival outcomes were evaluated in GSE65858.

Table S3. Candidate gene coverage and alias mapping.

| Coverage | Number of genes |
|---|---|
| Unique candidate genes | 493 |
| Measured in TCGA HNSC | 453 (442 direct, 11 via alias) |
| Measured in GSE65858 | 442 (433 direct, 9 via alias) |
| Measured in both cohorts; frozen pool | 414 |

Table S3 (continued). Symbols used to retrieve the 12 genes requiring an alias in at least one cohort.

| Candidate gene | TCGA HNSC symbol | GSE65858 symbol |
|---|---|---|
| CAVIN1 | PTRF | PTRF |
| CCN1 | CYR61 | CYR61 |
| CCN5 | WISP2 | WISP2 |
| ERO1A | ERO1L | ERO1L |
| MRGBP | MRGBP | C20orf20 |
| CADM1 | TSLC1 | CADM1 |
| CAVIN3 | PRKCDBP | PRKCDBP |
| CCN2 | CTGF | CTGF |
| DDR1 | NEP | DDR1 |
| NOCT | CCRN4L | CCRN4L |
| SIRPA | PTPNS1 | SIRPA |
| UTP11 | UTP11L | UTP11L |

The full 493 gene coverage and source mapping is provided as Data S1 (full_candidate_pool_final_coverage.csv). The 414 genes measurable in both cohorts formed the frozen candidate pool. A direct match uses the candidate symbol; an alias uses the displayed historical symbol. This table reports the supplied cross platform mapping, not a fresh nomenclature adjudication.
